# The Effect of Male Nurses’ Personality Traits, Perception of the Profession, and Job Motivation on Their Intentions to Quit: A Cross-Sectional Study

**DOI:** 10.3390/healthcare14070871

**Published:** 2026-03-28

**Authors:** Nukhet Bayer, Ayşegül Turan

**Affiliations:** 1 Nursing Department, Faculty of Health Sciences, Lokman Hekim University, 06510 Ankara, Turkey; nukhet.bayer@lokmanhekim.edu.tr; 2 Nursing Department, Faculty of Health Sciences, Kırşehir Ahi Evran University, 40100 Kırşehir, Turkey

**Keywords:** male nurses, personality traits, professional perception, job motivation, intention to quit

## Abstract

**Highlights:**

**What are the main findings?**

- Attitudes toward the nursing profession are the strongest negative predictor of intention to quit among male nurses.
- Personality features and job motivation are significantly associated with lower intention to quit, independent of demographic characteristics.

**What are the implications of the main findings?**

- Clinical and managerial initiatives aimed at strengthening professional identity and job motivation may contribute to reducing intention to quit among male nurses.
- Role model–based mentoring and motivation-enhancing training programs may support workforce retention and stability in clinical nursing settings.

**Abstract:**

Objective: This study aimed to examine the effects of personality features and attitudes toward the nursing profession on job motivation and intention to quit among male nurses within the framework of the Job Demands–Resources (JD-R) model. In this framework, personality traits and perceptions of the profession were conceptualized as personal resources, while job motivation represents a motivational process that may influence turnover intention. Methods: A cross-sectional design was employed with 303 male nurses actively working in different regions of Turkey. Data were collected via an online survey using non-probability sampling methods. The measurement tools included the Attitude Scale Toward the Nursing Profession, Job Motivation Scale, Personality Features Scale, and the Nurse Turnover Intention Scale. Data were analyzed using descriptive statistics, confirmatory factor analysis, and structural equation modeling. Results: Structural equation modeling revealed that attitudes toward the nursing profession (β = −0.90, *p* < 0.001), personality features (β = −0.10, *p* < 0.001), and job motivation (β = −0.14, *p* < 0.001) had significant and negative effects on intention to quit. Attitudes toward the profession emerged as the strongest predictor, explaining 49% of the variance in intention to quit. Attitudes toward the nursing profession, personality features, and job motivation were found to have significant and negative effects on intention to quit among male nurses. Consistent with the JD-R model, the findings suggest that personal resources (personality and professional perception) and motivational processes (job motivation) may play an important role in shaping turnover intentions among male nurses. Accordingly, professional identity-strengthening initiatives, role model-based mentoring, and motivation-enhancing training programs may help support the retention of male nurses in the profession.

## 1. Introduction

The nursing profession plays a pivotal role in the processes of preserving, promoting, and restoring human health. Historically, nursing has been strongly associated with characteristics such as care, compassion, and emotional labor, often being perceived as a profession predominantly for women [1]. However, in contemporary times, the nursing profession has transcended gender barriers, becoming an area where men also make significant contributions. While the number of male nurses is steadily increasing, this evolution also brings forth various challenges and professional adaptation processes.

Societal perceptions are considered a significant factor limiting men’s inclination toward the nursing profession. Male nurses entering the profession may encounter various role conflicts and professional difficulties in their working lives [2]. The literature indicates that male nurses are frequently assigned to units with physically and psychologically more intense workloads, such as emergency departments or intensive care units, work more night shifts, and may be excluded from certain clinical opportunities [3,4]. Such experiences can deeply affect male nurses’ perceptions of the profession, their motivation, and organizational commitment. In this context, understanding male nurses’ intentions to change jobs becomes crucial for their professional retention and sustainability.

One of the most widely used theoretical frameworks to explain the relationships between employees’ job demands and resources in the work environment, and their psychological and behavioral outcomes, is the Job Demands–Resources (JD-R) model. The JD-R model explains employees’ experiences in their work life through two core components: job demands and job resources [3,5]. Job demands refer to elements requiring physical, cognitive, or emotional effort from employees to fulfill their tasks, while job resources encompass factors that facilitate employees’ achievement of work goals, support their personal development, and enhance their motivation [3]. This model specifically suggests that job resources increase employee motivation, leading to positive organizational outcomes; conversely, a lack of resources is associated with negative outcomes such as stress, burnout, and intention to quit.

The JD-R model considers personal resources as an important explanatory factor in addition to organizational resources. Personal resources are defined as psychological attributes that enable individuals to cope with environmental demands and achieve their goals. In this scope, an individual’s personality traits and attitudes towards the profession are considered important personal resources [6]. Personality traits are fundamental psychological structures that influence how an individual perceives and reacts to stressors in the work environment. Similarly, positive attitudes towards the profession can strengthen employees’ professional commitment and motivation, whereas negative attitudes may lead to decreased job satisfaction and an increased tendency to leave the profession. Studies in the literature demonstrate that personality traits have significant effects on job motivation and professional commitment [7,8].

Job motivation is considered one of the key mechanisms linking job resources with organizational outcomes within the JD-R model. Employees with high job motivation exhibit greater commitment to their work, enhance their performance, and have a lower propensity to leave the organization. Research conducted on nurses reveals that job motivation is closely associated with intention to quit through variables such as job satisfaction and organizational commitment. Smokrovic et al. [7] indicated that job motivation indirectly affects intention to quit through job satisfaction in nurses. Similarly, Çeliktürk Doruker et al. [9] showed that positive perceptions of the work environment increase career motivation, thereby enhancing job satisfaction and reducing intention to quit. Another study conducted in Turkey found that job motivation mediates the relationship between career barriers and intention to quit [10].

An examination of the existing literature focusing on male nurses reveals that studies largely concentrate on organizational factors such as organizational support, working conditions, or perceptions of professional prestige [10,11,12]. In contrast, studies examining the holistic effects of personal resources, such as male nurses’ personality traits and attitudes towards the profession, on job motivation and intention to quit are quite limited. Furthermore, empirical studies on how the JD-R model operates in the context of male nurses to explain the relationships between personal resources, motivational processes, and intention to quit are also found to be insufficient. This indicates a significant research gap in the literature. To address this gap, this study aims to investigate the effects of male nurses’ personality traits, attitudes toward the profession, and job motivation on their intentions to change jobs, based on the Job Demands–Resources (JD-R) model. In this framework, it is hypothesized that job motivation may act as an explanatory mechanism in the relationship between personal resources and intention to quit.

### Conceptual Framework

The nursing profession has historically been associated with characteristics such as care, compassion and emotional labour, and has therefore been perceived for many years as a profession synonymous with women [1]. This societal perception is considered a significant factor limiting men’s entry into the nursing profession; male nurses who do enter the profession may face various role conflicts and professional challenges in their working lives [2]. The literature indicates that male nurses are frequently assigned to units with a heavier physical and psychological workload, such as A&E or intensive care, work more night shifts, and may be excluded from certain clinical opportunities [3,4]. Such experiences can influence male nurses’ perceptions of the profession, their motivation, and their organisational commitment.

In this context, one of the most widely used theoretical frameworks for explaining the relationships between job demands and resources in the workplace and their psychological and behavioural consequences is the Job Demands–Resources (JD-R) model. The JD-R model is based on two core components—job demands and job resources—to explain employees’ experiences in the workplace [3,5]. Job demands are defined as elements that require physical, cognitive or emotional effort for employees to perform their duties and can lead to negative outcomes such as burnout in the long term. In contrast, job resources refer to factors that facilitate employees’ achievement of work goals, support their personal development, and enhance their motivation [3]. The JD-R model posits that job resources, in particular, lead to positive organisational outcomes by increasing employee motivation; conversely, a lack of resources is associated with negative outcomes such as stress, burnout, and intention to leave.

The JD-R model treats organisational resources and personal resources as explanatory factors. Personal resources are defined as the psychological characteristics that enable individuals to cope with environmental demands and achieve their goals. In this context, an individual’s personality traits and attitudes towards their profession are considered important personal resources [6]. Personality traits are important psychological constructs that influence how an individual perceives stress factors in the workplace and how they respond to such situations. Similarly, positive attitudes towards the profession can strengthen employees’ commitment and motivation, whilst negative attitudes may lead to reduced job satisfaction and an increased tendency to leave the job.

Studies in nursing literature indicate that personality traits have a significant impact on work motivation and professional commitment [7,8]. An individual’s positive personality traits can enhance motivation levels by enabling them to cope more effectively with challenges in the workplace. Conversely, negative attitudes towards the profession or a lack of alignment with professional values can weaken employees’ motivation and increase their intention to leave. Therefore, within the context of the JD-R model, personality traits and attitudes towards the profession can be regarded as important personal resources influencing employees’ motivation processes.

Job motivation is recognised as one of the key mechanisms linking job resources to organisational outcomes in the JD-R model. Employees with high job motivation demonstrate greater commitment to their work, improve their performance, and are less likely to leave the organisation. Studies conducted on nurses in the literature reveal that job motivation is closely related to intention to quit through variables such as job satisfaction and organisational commitment. In a study conducted by Smokrovic and colleagues [7] on nurses, it was determined that job motivation indirectly influences intention to quit via job satisfaction. Similarly, Celikturk Doruker and colleagues [9] demonstrated that positive perceptions of the work environment increase career motivation, thereby enhancing job satisfaction and reducing intention to leave. In a study conducted in Turkey, it was found that work motivation acts as a mediator in the relationship between career barriers and intention to leave [10].

A review of studies focusing on male nurses reveals that the literature largely concentrates on organisational factors (such as organisational support, working conditions or perceptions of professional prestige) [10,11,12]. In contrast, studies examining the holistic effects of male nurses’ personal resources—such as personality traits and attitudes towards the profession—on work motivation and intention to leave are quite limited. Furthermore, it is evident that empirical studies examining how the JD-R model operates to explain the relationships between personal resources, motivational processes and intention to leave in the context of male nurses are also limited. This situation points to a significant research gap in the literature.

In this study, with the aim of addressing this gap, the effects of male nurses’ personality traits and attitudes towards the profession on their work motivation and intention to leave are examined, based on the JD-R model. Within this framework, it is hypothesised that work motivation may act as an explanatory mechanism in the relationship between personal resources and intention to leave. In line with this theoretical framework, the following hypotheses have been formulated:

**H1.**  *Male nurses’ personality traits influence their intention to leave their jobs.*

**H2.**  *Male nurses’ attitudes towards their profession influence their intention to leave the profession.*

**H3.**  *Male nurses’ job motivation influences their intention to leave their jobs.*

## 2. Method

### 2.1. Study Design and Participants

This study is designed as a descriptive and cross-sectional study aimed at examining the effects of personality traits, attitudes towards the profession and work motivation on the intention to leave the profession among male nurses. The cross-sectional study design is considered a suitable approach for examining the relationships between variables at a specific point in time. The population of the study consists of male nurses currently practising in Turkey. As there is no up-to-date and accessible data on the exact number of male nurses working in Turkey, non-probability sampling methods were used in the selection of the sample. In this context, convenience sampling and snowball sampling techniques were applied in conjunction.

During the data collection process, male nurses working in various provinces across Turkey were contacted via social and professional networks, and an online survey form was distributed. Participants were asked to share the survey link with their colleagues, thereby supporting the snowball sampling method. This approach is frequently used in research targeting hard-to-reach or specific professional groups. However, non-probability sampling methods may limit the sample’s ability to represent the entire population and increase the risk of selection bias. Online data collection methods may lead to individuals with access to digital platforms or those more willing to participate in the research being over-represented in the sample. Therefore, the study’s findings should be interpreted with caution when directly generalising them to all male nurses in Turkey.

G*Power 3.1.9.7. software was used to determine the minimum required sample size for the study [13]. Based on the planned analyses and the variables included in the study, the power analysis determined that a minimum of 119 participants was required to achieve 95% statistical power (α = 0.05) and a medium effect size (f^2^ = 0.159). The data collection instrument used in the study consists of a total of 65 items [14]. In structural equation modelling studies, it is recommended that a minimum of 4 participants be included for each item. Accordingly, 303 male nurses were recruited for the study, and the analyses were conducted using this sample. Inclusion criteria for the study were: being male, actively working as a nurse in a healthcare institution, having at least six months of professional experience, being over 18 years of age, and voluntarily participating in the study. Participants who did not complete the questionnaire in full and those who stated they were not currently working as nurses were excluded from the study. Data were collected between January and February 2025.

### 2.2. Data Collection Tool

The data collection tool consisted of two parts: 

Personal Information Form:

This form comprises questions about the individual and professional characteristics of nurses.

Job Motivation Scale: 

Job motivation was measured in two sub-dimensions: work motivation and family motivation. The scale consists of 5 items and is a 5-point Likert-type (1 = Strongly Disagree… 5 = Strongly Agree). High scores obtained from the scale indicate that the individual has high motivation to continue their job. In the original study of the scale, it was reported that the construct validity and internal consistency were at an acceptable level, and that it showed significant relationships with organizational outcomes such as intention to quit and job commitment [15]. The reliability coefficient of the job motivation scale in our study is 0.67.

Attitude Scale Towards the Nursing Profession: 

The Attitude Scale Towards the Nursing Profession, developed by Çoban [16], consists of 40 items. It comprises three sub-dimensions: characteristics of the nursing profession, preference for the nursing profession, and attitude towards the general state of the nursing profession. The Cronbach’s Alpha coefficient of the scale was found to be 0.91. The reliability coefficient of The Attitude Scale Towards the Nursing Profession in our study is 0.96.

Turnover Intention Scale for Nurses:

The 10-item scale developed by Yeun and Kim [17] was adapted into Turkish by Zeyrek et al. [18]. The scale, which is a five-point Likert-type (1 = Strongly Disagree … 5 = Strongly Agree), has a single-factor structure and high internal consistency (Cronbach alpha = 0.902). The reliability coefficient of the Turnover Intention Scale in our study is 0.88.

Personality Features Scale:

The Turkish adaptation and validity and reliability study of the scale developed by Gosling et al. [19] was carried out by Atak [20]. The scale consists of 10 items and comprises five sub-dimensions: “Extroversion, Openness to Experience, Conscientiousness, Agreeableness, and Emotional Stability”. The scale is a 5-point Likert-type scale (1 = Strongly Disagree, …, 5 = Strongly Agree), and the Cronbach’s alpha internal consistency coefficients for the sub-dimensions were found to be between 0.81 and 0.86. The reliability coefficient of the Turnover Intention Scale in our study is 0.76.

### 2.3. Validation of the Validity and Reliability of Scales

Confirmatory Factor Analysis (CFA) and internal consistency analyses were conducted to verify the validity and reliability of the scales used in the study on the current sample. CFA analyses were applied to assess whether the theoretically proposed factor structures of the scales were confirmed in the research sample. In evaluating model fit, the fit indices commonly used in the literature (χ^2^/df, CFI, TLI, RMSEA and SRMR) were considered. Furthermore, the internal consistency of the scales was assessed using Cronbach’s alpha coefficients.

### 2.4. Data Analysis

The research was analysed using SPSS 29 and AMOS 24 software packages. Descriptive statistics included mean, standard deviation, frequency, and percentage calculations, while internal consistency analyses and Confirmatory Factor Analysis (CFA) were applied to verify the factor structure of the scales. Structural Equation Modeling (SEM) was used to test the effects of independent variables on intention to quit.

## 3. Results

A total of 303 male nurses took part in the study. The mean age of the participants was 32.41 ± 8.39. 29.0% of the participants were aged between 26 and 30, whilst 30.7% were aged 36 and over. In terms of professional experience, it was found that approximately half of the participants (49.5%) had between 1 and 5 years’ work experience. When assessed by department, a significant proportion of the participants were found to be working in the intensive care unit (40.9%). Regarding working patterns, 54.5% of the nurses were found to be working on a shift system. Most participants hold a bachelor’s degree (71.0%). It was found that 52.1% of participants are married and 43.2% have children. Detailed information regarding the participants’ socio-demographic characteristics is presented in Table 1.

### 3.1. Descriptive Findings Regarding the Scales

Descriptive statistics and reliability analyses were conducted for the scales used in the study. Firstly, skewness and kurtosis values were examined to assess whether the data set followed a normal distribution. It was observed that the skewness and kurtosis values for all variables fell within the range of −1.5 to +1.5, and it was accepted that the data met the assumption of normal distribution [21]. The internal consistency of the scales was assessed using Cronbach’s alpha. The alpha values of the scales ranged from 0.67 to 0.96. The scale measuring attitudes towards the nursing profession (α = 0.96) and the scale measuring intention to leave the job (α = 0.88) demonstrated high levels of internal consistency. The reliability of the personality traits scale was found to be at an acceptable level (α = 0.76). Although Cronbach’s alpha value of the work motivation scale (α = 0.67) is close to the 0.70 threshold recommended in the literature, it is known that the alpha coefficient can be relatively low in short scales [22]. For this reason, the scale’s factor structure was additionally tested using confirmatory factor analysis. The mean scores for the scales range from 2.83 ± 0.70 to 3.80 ± 0.68. Descriptive statistics for the scales are presented in Table 2.

### 3.2. Confirmatory Factor Analysis of Scales

Confirmatory Factor Analysis (CFA) was conducted to validate the factor structures of the scales used in the study on the current sample. To assess model fit, the χ^2^/df, RMSEA, GFI, CFI and NFI fit indices were examined. According to the results obtained:

The model fit for the Attitude Scale towards the Nursing Profession is at an acceptable level (χ^2^/df = 2.85, RMSEA = 0.07, CFI = 0.94).

The Work Motivation Scale demonstrates good model fit (χ^2^/df = 1.57, RMSEA = 0.04, CFI = 0.98).

The Intent to Leave Scale exhibits strong model fit indices (χ^2^/df = 1.78, RMSEA = 0.05, CFI = 0.99).

The Personality Traits Scale also demonstrates an acceptable level of fit (χ^2^/df = 2.33, RMSEA = 0.06, CFI = 0.97).

An examination of the fit indices for the structural model developed in the study revealed the following values: χ^2^/df = 3.19, RMSEA = 0.08, CFI = 0.88 and NFI = 0.84. These values indicate that the model demonstrates an acceptable level of fit. In general, it can be stated that the factor structures of the scales demonstrate acceptable and good model fit within the study sample. The CFA findings regarding the scales are presented in Table 3.

### 3.3. Effects of Perception of Nursing Profession, Personality Features, and Job Motivation on Intention to Quit

**Figure 1:**
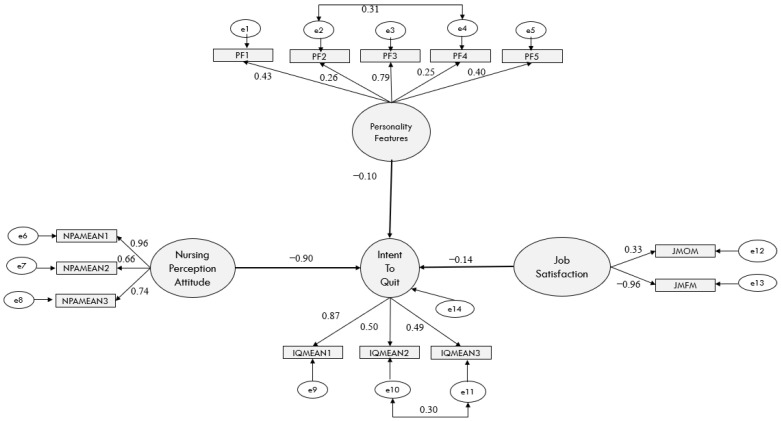
The effects of perception of the nursing profession, personality features, and job motivation on intention to quit: a structural equation model.

The effects of perception of nursing profession, personality features, and job motivation on intention to quit of male nurses were examined using structural equation modeling analysis. Perception of nursing profession, personality features, and job motivation negatively and significantly affect the intention to quit (Table 4, Figure 1).

According to these findings:

Attitudes towards the nursing profession have a strong and negative effect on the intention to leave the profession (β = −0.90, *p* < 0.001).

Personality traits have a negative and statistically significant effect on intention to leave (β = −0.10, *p* < 0.001).

Work motivation has a negative and significant effect on the intention to leave (β = −0.14, *p* < 0.001).

When effect sizes were assessed in accordance with the criteria proposed by Cohen [23]:

It is observed that the effect of attitude towards the nursing profession on intention to leave is substantial (f^2^ = 0.96), that personality traits have a moderate effect (f^2^ = 0.33), and that job motivation has a small but significant effect (f^2^ = 0.09). In the model, the variable representing attitude towards the nursing profession explains 49% of the variance in intention to leave, whilst personality traits explain 25% and work motivation explains 9%. Based on these findings, hypotheses H1, H2 and H3 have been accepted.

## 4. Discussion

This study aimed to examine the effects of attitudes towards the profession, personality traits and work motivation on intention to quit among male nurses. The results of the structural equation modelling indicate that attitudes towards the nursing profession have a strong and negative effect on intention to quit. This finding is consistent with previous studies indicating that professional identity and positive perceptions of the profession are decisive factors in employees’ intention to remain in their jobs [24]. Indeed, the literature reports that strengthening professional identity increases nurses’ commitment to their profession and enhances the likelihood of them remaining in their jobs [25]. These findings become more meaningful when evaluated within the framework of the Job Demands–Resources (JD-R) model. According to the JD-R model, the balance between the demands employees face in the workplace and the resources they possess determines their motivational processes and organisational outcomes. In this model, personal resources are regarded as key factors that mitigate the negative effects of job demands and support motivational processes. The professional attitudes and personality traits examined in this study can be considered important personal resources for male nurses. Particularly for male nurses working in environments with high job demands, such as intensive care, emergency departments or shift work, it can be argued that positive professional attitudes and strong personality traits may play a protective or buffering role against the intention to leave the profession.

One of the study’s notable findings is that the impact of attitudes towards the profession on the intention to leave the job is quite strong. This situation can be assessed from several conceptual perspectives. Firstly, as the nursing profession has historically been associated with women due to gender roles, male nurses’ perceptions of the profession and the development of their professional identity may be subject to different dynamics. In this context, it is conceivable that male nurses who fail to develop a positive attitude towards the profession may experience a weakening of their professional commitment, which could in turn increase their intention to leave the profession. Indeed, studies conducted in Turkey indicate that male nurses may place less importance on professional values compared to female nurses and may exhibit lower levels of positive attitudes towards the profession [26,27,28,29]. However, findings regarding gender differences in the international literature appear to be more inconsistent; whilst some studies find no significant gender-related differences, others suggest that female nurses may have more positive attitudes towards the profession [30,31].

The strong influence of attitudes towards the profession also demonstrates that male nurses’ professional identity and sense of belonging play a critical role in their intention to leave the profession. This situation highlights that the factors influencing male nurses’ retention in the profession are closely linked not only to organisational conditions but also to the social perception of the profession and individual professional identity. Furthermore, the literature emphasises that factors such as economic responsibilities, family support and childcare obligations may also influence male nurses’ decision to remain in the profession. Indeed, a study conducted with male nurses reported that gender role conflict and intention to leave the profession did not show significant differences according to certain professional variables such as years of service, work unit or shift [32].

Another key finding of the study is that personality traits have a negative and significant effect on the intention to leave. When assessed within the context of the JD-R model, personality traits are among the key personal resources that enhance an individual’s capacity to cope with stressful work conditions. In particular, personality traits such as extraversion, conscientiousness and emotional stability can support motivation processes by making it easier for employees to cope with job demands. It can therefore be argued that nurses with strong personal traits may be able to manage job stress more effectively and, consequently, have a lower intention to leave their jobs.

The study also found that job motivation has a negative and significant effect on the intention to leave. According to the JD-R model, motivational processes are one of the fundamental mechanisms that enable employees to enhance their performance and job commitment by utilising job resources. Job motivation can help nurses find meaning in their work and strengthen their commitment to their professional goals. In this context, it can be said that motivational sources such as job motivation and family motivation play a significant role in nurses’ decisions to remain in their jobs. The literature reports that as motivation levels increase among nurses, job performance rises and the intention to leave the job decreases [11]. Similarly, international studies indicate that as professional identity and psychological resources increase, the intention to leave the profession decreases significantly [33]. Furthermore, it has been reported that individual resources—such as self-efficacy, optimism and psychological resilience, which are components of psychological capital—are negatively associated with the intention to leave the profession [34,35].

Overall, the findings of this study are consistent with the predictions of the JD-R model. It appears that personal resources, such as personality traits and attitudes towards the profession, support motivational processes among male nurses in a healthcare environment characterised by high job demands, and play a significant role in reducing their intention to leave the profession. These findings suggest that policies aimed at retaining male nurses should not be limited to focusing solely on improving working conditions but should also incorporate psychosocial dimensions such as the development of professional identity, the reinforcement of positive attitudes towards the profession, and the development of motivational support mechanisms.

### 4.1. Limitations of the Study

When interpreting the findings of this study, certain methodological limitations must be taken into account. Firstly, the data for this study were collected using an online survey method. During the data collection process, the survey link was distributed to male nurses working in various provinces across Turkey via the researchers’ academic and professional networks, and participants were asked to share the link with their colleagues. In this process, nurses were specifically reached via professional communication groups, social media platforms and professional networks. However, this approach may increase the risk of selection bias, as it could lead to individuals with greater online access or a greater willingness to participate in the study being over-represented in the sample.

Although the convenience sampling and snowball sampling methods used in the study facilitate reaching specific occupational groups, the fact that they are non-probability sampling techniques may limit the sample’s ability to fully represent all male nurses working in Turkey. Therefore, caution should be exercised when generalising the study’s findings to the entire male nursing population. However, the inclusion of participants from different cities and various clinical units contributes to a certain degree of diversity within the sample.

Another limitation of the study is its cross-sectional research design. Whilst cross-sectional studies allow for the examination of relationships between variables within a specific time frame, they do not permit the establishment of causal relationships between variables. Consequently, whilst the findings of this study explain the relationships between personality traits, attitudes towards the profession, work motivation and intention to leave the job, they do not allow for definitive conclusions regarding the causal nature of these relationships.

The use of longitudinal or experimental research designs in future studies could provide a more comprehensive understanding of changes in male nurses’ professional motivation over time and the dynamics of their intention to leave the profession. Furthermore, comparative studies conducted in different countries or within different healthcare system contexts could contribute to a broader assessment of the factors influencing male nurses’ professional experiences.

### 4.2. Theoretical Contributions

This study makes significant theoretical contributions to research in the nursing literature, examining the factors explaining intention to leave. Firstly, the study demonstrates that intention to leave is associated with factors relating to the organisational or work environment, as well as with individual psychological resources. When the findings obtained in this context are evaluated within the framework of the Job Demands–Resources (JD-R) model, they reveal that individual characteristics such as personality traits, attitudes towards the profession and work motivation can function as personal resources. According to the JD-R model, personal resources can strengthen the tendency to remain in employment by increasing individuals’ resilience against the stress and risk of burnout caused by high job demands. In this study, the particularly strong effect of attitude towards the profession on intention to leave suggests that professional identity and professional belonging may play a central role in explaining nurses’ retention behaviour.

Another theoretical contribution of this study is its focus on male nurses. Whilst the intention to leave the profession has mostly been examined in the nursing literature within the general nursing population, the professional experiences and motivational processes of male nurses have been addressed in only a limited number of studies. By examining how individual factors such as attitudes towards the profession, personality traits and motivation relate to intention to leave among male nurses, this study contributes to filling this gap in the literature. In particular, the strong influence of attitudes towards the profession suggests that male nurses’ professional identity development and perceptions of the profession may be a critical factor in their decisions to remain in the profession.

The study examined the multidimensional nature of motivation by considering both the work motivation and family motivation dimensions together. This approach demonstrates that it is not only individuals’ motivation related to the work environment that influences their career decision-making processes but also factors such as family responsibilities and social roles. In this respect, the study offers a perspective that expands the personal resources dimension of the JD-R model by evaluating both individual psychological characteristics and social motivational factors together.

### 4.3. Practical Implications

The findings of this study offer important practical implications for healthcare organisations and nursing managers. The findings indicate that male nurses’ attitudes towards the profession are a strong predictor of their intention to leave the profession. This suggests that interventions aimed at strengthening nurses’ professional identity may support workforce retention. Within healthcare organisations, career development programmes, the implementation of professional role models, and educational activities that reinforce professional values may be effective in enhancing male nurses’ positive attitudes towards the profession.

The fact that individual resources such as personality traits and work motivation are associated with the intention to leave suggests that healthcare organisations should not only focus on workload and working conditions, but also develop management strategies that strengthen staff’s psychological resources. In this context, leadership support, management practices that increase staff engagement, and programmes that strengthen psychological resilience can boost nurses’ motivation and thereby reduce their intention to leave.

The study demonstrates that when considering the work and family dimensions of job motivation, nurses’ needs regarding work–life balance must also be taken into account. In particular, supporting a balance between shift work patterns, family responsibilities and social life can enhance nurses’ professional commitment. In this context, flexible working arrangements, family-friendly organisational policies and employee support programmes are among the key measures that can strengthen nurses’ retention.

Overall, the findings of this study suggest that strategies aimed at reducing nurses’ intention to leave should focus on reducing work demands and strengthening staff’s personal and motivational resources. This approach could make a significant contribution to sustainable human resources management and the continuity of high-quality care services within healthcare organisations.

## 5. Conclusions

This study demonstrates that individual factors such as personality traits, attitudes towards the profession and work motivation are significantly associated with intention to leave among male nurses. The findings suggest that attitudes towards the profession are strongly associated with intention to leave. Furthermore, personality traits and work motivation were also found to be significantly, though to a relatively lesser extent, associated with intention to leave. These results suggest that individual psychological resources and motivational processes may play an important role in male nurses’ career decision-making processes.

When the findings are evaluated within the framework of the Job Demands–Resources (JD-R) model, it can be concluded that individual factors such as attitudes towards the profession, personality traits and motivation may function as personal resources that facilitate nurses’ ability to cope with demands in their working lives. In this context, strengthening personal resources may play a role in reducing the intention to leave in healthcare settings characterised by high job demands. However, given the cross-sectional design of the study, it would be more appropriate to assess the identified relationships at a correlational level rather than interpreting them within a causal framework.

The findings of the study also offer some practical implications for nursing management. Firstly, strengthening male nurses’ attitudes towards the profession can be considered a potential area that could help reduce their intention to leave the profession. In this regard, healthcare institutions could plan mentoring programmes, role model initiatives and training activities aimed at reinforcing professional values to support the development of professional identity. Furthermore, developing management practices that support the career development of nurse managers, the sharing of clinical responsibilities, and equal access to opportunities for professional advancement could help strengthen male nurses’ professional commitment.

Leadership approaches that support employees’ motivational resources, management practices that encourage employee engagement, and corporate policies focused on work–life balance can also help retain nurses. In particular, within healthcare organisations where shift work is prevalent, practices that support employees’ psychological and social resources can help boost job motivation.

Further research involving different samples is required to validate the findings of this study more comprehensively. Future longitudinal studies could provide a more detailed picture of how the relationships between male nurses’ professional attitudes, motivational processes and intention to leave the profession change over time. Furthermore, comparative studies conducted in different cultural contexts could contribute to a broader assessment of the factors influencing male nurses’ professional experiences.

## Figures and Tables

**Table 1:** Socio-demographic characteristics of participants (N:303).

Age X: 32.41 Sd: 8.39	N	%	Working Year	N	%
25 and under	77	25.4	1–5 Years	150	49.5
26–30	88	29.0	6–10 Years	50	16.5
31–35	45	14.9	11–15 Years	59	19.5
36 and over	93	30.7	16 Years and over	44	14.5
**Working Time in the Institution**	**N**	**%**	**Working Position**	**N**	**%**
1–5 Years	204	67.3	Charge Nurse	46	15.2
6–10 Years	35	11.6	Service Nurse	181	59.7
11 Years and over	64	21.1	Other	76	25.1
**Working Type**			**Educational Status**		
Always working daytime	68	22.4	Health Vocational High School	31	10.2
Always working at night	27	8.9	Associate degree	38	12.5
Shift work	165	54.5	Bachelor’s degree	215	71.0
Other	43	14.2	Postgraduate	19	6.3
**Marital status**	**N**	**%**	**Having children**	**N**	**%**
Married	158	52.1	Yes	131	43.2
Single	145	47.9	No	172	56.8
**Unit worked in**			**City**		
General Surgery	31	10.2	Ankara	153	50.5
Intensive Care	124	40.9	Bayburt	5	1.7
Administrative Unit	12	4.0	Bursa	5	1.7
Emergency Room	32	10.6	Erzincan	12	4.0
Children’s Service	6	2.0	Giresun	4	1.3
Cardiology Department	3	1.0	Hatay	4	1.3
Operating Room	13	4.3	İstanbul	38	12.5
Angiography Unit	14	4.6	İzmir	32	10.6
Urology Service	4	1.3	Kars	4	1.3
Psychiatry Service	3	1.0	Kayseri	16	5.3
Chest Diseases	10	3.3	Osmaniye	4	1.3
Oncology Service	5	1.7	Samsun	4	1.3
Palliative Service	14	4.6	Şanlıurfa	4	1.3
Dialysis Unit	5	1.7	Tokat	6	2.0
Gastroenterology	3	1.0	Antalya	12	4.0
Home Healthcare Services	5	1.7			
Vaccination Programming	3	1.0			
Internal Diseases	16	5.3			

**Table 2:** Descriptive findings for the scales.

	Score	Average	Standard Deviation	Skewness	Kurtosis	Cronbach Alfa
NPAMEAN	32.82	2.31	0.85	−0.26	−0.08	0.96
IQMEAN	66.55	3.66	0.83	−0.362	−0.130	0.88
JMMEAN	46.81	2.87	0.57	−0.352	0.447	0.67
PFMEAN	31.52	2.26	0.66	0.455	−0.039	0.76

Note: NPAMEAN: Nursing perception attitude overall mean, IQMEAN: Intention to quit scale overall mean, JMMEAN: Job motivation overall mean, PFMEAN: Personality features scale overall mean.

**Table 3:** Confirmatory factor analysis findings of the scales.

	X^2^/DF	RMSEA	GFI	CFI	NFI
Perception of Nursing Profession Scale	2.85	0.07	0.85	0.94	0.91
Job Motivation Scale	1.57	0.04	0.97	0.98	0.99
Intention to Quit Scale	1.78	0.05	0.96	0.99	0.98
Personality Features Scale	2.33	0.06	0.96	0.97	0.96
SEM	3.19	0.08	0.92	0.88	0.84

**Table 4:** Effects of nursing perception attitude, personality features, and job motivation on intention to quit—SEM coefficients.

Nursing perception attitude	→	Intention to quit	**Standardize Β**	**Standard deviation**	**t**	***p***
			−0.90	0.07	7.85	***
Personality features	→	Intention to quit	−0.10	0.05	1.98	***
Job motivation	→	Intention to quit	−0.14	0.07	2.80	***
**Independent Variables**	**R^2^**	**1 − R^2^**	***F*^2^ (R^2^/1 − R^2^)**			
Nursing perception attitude	0.49	0.51	0.96			
Personality features	0.25	0.75	0.33			
Job motivation	0.09	0.91	0.09			

***: *p* ≤ 0.001.

## Data Availability

The data that support the findings of this study are available from the corresponding author upon reasonable request.

## Abbreviations

The following abbreviations are used in this manuscript:

CFA	Confirmatory Factor Analysis
CFI	Comparative Fit Index
DF	Degree of Freedom
GFI	Goodness-of-Fit Index
IQMEAN	Intention to quit scale overall mean
JD-R	Job Demands-Resources
JMMEAN	Job motivation overall mean
NPAMEAN	Nursing perception attitude overall mean
PFMEAN	Personality features scale overall mean
RMSEA	Root Mean Square Error of Approximation
SEM	Structural Equation Modeling

## References

[1] Bayer N., Golbası Z. (2021). Experiences of men in nursing: A qualitative study of perspectives of nurses. Era’s J. Med. Res..

[2] Chang H.E., Jeong S. (2021). Male nurses’ experiences of workplace gender discrimination and sexual harassment in South Korea: A qualitative study. Asian Nurs. Res..

[3] Shen J., Guo Y., Chen X., Tong L., Lei G., Zhang X. (2022). Male nurses’ work performance: A cross-sectional study. Medicine.

[4] Ng M., See C., Ignacio J. (2024). Qualitative systematic review: The lived experiences of males in the nursing profession on gender discrimination encounters. Int. Nurs. Rev..

[5] Kim S.O., Moon S.H. (2021). Factors influencing turnover intention among male nurses in Korea. Int. J. Environ. Res. Public Health.

[6] Bakker A.B. (2015). A job demands–resources approach to public service motivation. Public Adm. Rev..

[7] Bakker A.B., Demerouti E. (2017). Job demands-resources theory: Taking stock and looking forward. J. Occup. Health Psychol..

[8] Deng J., Wang P., Tian X., Li K., Yang L., Ding S. (2024). Turnover intention and its influencing factors among male nurses in China: A national-scale descriptive study. BMC Nurs..

[9] Kim I.J., Shim H.W. (2018). Subjectivity about turnover intention among male nurses in South Korea: A Q-methodological study. Asian Nurs. Res..

[10] Smokrovic E., Kizivat T., Bajan A., Solic K., Gvozdanovic Z., Farcic N., Zvanut B. (2022). A conceptual model of nurses’ turnover intention. Int. J. Environ. Res. Public Health.

[11] Celikturk Doruker N., Hacioglu G., Nurulke B., Ceylan L. (2025). Investigation of the relationship between work motivation, work performance and turnover intention of surgical nurses: A cross-sectional study. Appl. Nurs. Res..

[12] Yeşilyurt T., Göktepe N., Polat Ş. (2023). The mediating effect of job motivation on the relationship between career barriers and nurses’ turnover intention. Collegian.

[13] Faul F., Erdfelder E., Lang A.-G., Buchner A. (2007). G*Power 3: A flexible statistical power analysis program for the social, behavioral, and biomedical sciences. Behav. Res. Methods.

[14] Ebadi A., Taghizadeh Z., Montazeri A., Shahvari Z., Tavousi M., Bagherzadeh R. (2017). Translation, development and psychometric properties of health-related measures-Part 2: Construct validity, reliability and responsiveness. Payesh.

[15] Menges J.I., Tussing D.V., Wihler A., Grant A.M. (2016). When job performance is all relative: How family motivation energizes effort and compensates for intrinsic motivation. Acad. Manag. J..

[16] Çoban G.İ. (2010). Development of an Attitude Scale Towards the Nursing Profession. Ph.D. Thesis.

[17] Yeun E.J., Kim H. (2013). Development and testing of a nurse turnover intention scale (NTIS). J. Korean Acad. Nurs..

[18] Zeyrek A., Fidan Ö., Köktaş N. (2023). The adaptation of the Nurse Turnover Intention Scale into Turkish: A validity and reliability study. Int. J. Nurs. Pract..

[19] Gosling S.D., Rentfrow P.J., Swann W.B. (2003). A very brief measure of the Big-Five personality domains. J. Res. Pers..

[20] Atak H. (2013). The Turkish adaptation of the ten-item personality inventory. Noro. Psikiyatr. Ars..

[21] Tabachnick B.G., Fidell L.S. (2013). Using Multivariate Statistics.

[22] Taber K.S. (2018). The use of Cronbach’s alpha when developing and reporting research instruments in science education. Res. Sci. Educ..

[23] Cohen J. (1988). Statistical Power Analysis for the Behavioral Sciences.

[24] Hanum A.L., Hu Q., Wei W., Zhou H., Ma F. (2023). Professional identity, job satisfaction, and intention to stay among clinical nurses during the prolonged COVID-19 pandemic: A mediation analysis. jpn J. Nurs. Sci..

[25] Zhong Y., Ma H., Zhang C.C., Jiang Q.Y., Li J., Liao C.J., Liang Y.F., Shu L. (2024). Professional identity, job satisfaction, and turnover intention among Chinese novice nurses: A cross-sectional study. Medicine.

[26] Şenol F., Ziyafet U. (2019). The determination of occupational professional attitude of nurses in different generations. Health Soc..

[27] Dığın F., Mercan Y., Soydan C., Cebeli Çelebi H., Tenay R. (2023). Predictors of professional values in male and female nurses. J. Health Nurs. Manag..

[28] Efteli E., Yaman A., Orun Kavak H. (2023). Determining the attitudes of nursing students towards the nursing profession. Turk. J. Health Sci. Life.

[29] Karadağ S., Kılıç Z., Ceyhan Ö., Şentürk A. (2014). Nurses’ professional values and affecting factors. J. Psychiatr. Nurs..

[30] Fernández-Feito A., Basurto-Hoyuelos S., Palmeiro-Longo M.R., García-Díaz V. (2019). Differences in professional values between nurses and nursing students: A gender perspective. Int. Nurs. Rev..

[31] Rekisso A.D., Mengistu Z., Wurjine T.H. (2022). Nurses’ attitudes towards the nursing profession and associated factors in selected public hospitals, Addis Ababa, Ethiopia, 2021: A cross-sectional study. BMC Nurs..

[32] Hwang H.M., Kim M.J. (2017). Relationship of gender role conflict and job satisfaction to turnover intention for men in nursing. J. Korean Acad. Nurs. Adm..

[33] Hu H., Wang C., Lan Y., Wu X. (2022). Nurses’ turnover intention, hope and career identity: The mediating role of job satisfaction. BMC Nurs..

[34] Bozma K., Çınar O. (2025). The impact of psychological capital on work stress and turnover intention in the health sector. Kafkas Üniversitesi İktisadi Ve İdari Bilim. Fakültesi Derg..

[35] Tosun B., Güner Kibaroğlu G. (2025). Reducing turnover intention through organizational trust and self-efficacy. Isarder.

